# *In vivo* bioluminescence imaging of locally disseminated colon carcinoma in rats

**DOI:** 10.1038/sj.bjc.6601637

**Published:** 2004-03-02

**Authors:** S Zeamari, G Rumping, B Floot, S Lyons, F A Stewart

**Affiliations:** 1 Divisions of Experimental Therapy, The Netherlands Cancer Institute/Antoni van Leeuwenhoek Hospital, Plesmanlaan 121, Amsterdam 1066 CX, The Netherlands; 2 Molecular Genetics, The Netherlands Cancer Institute/Antoni van Leeuwenhoek Hospital, Plesmanlaan 121, Amsterdam 1066 CX, The Netherlands

**Keywords:** bioluminescence, *in vivo* imaging, luciferase, CC531 colorectal carcinoma, chemotherapy

## Abstract

Animal tumour models using orthotopic tumours for the evaluation of cancer therapies are of greater clinical relevance than subcutaneous models, but they also pose greater difficulties for measuring tumour size and quantifying response to treatment. In this study, we used noninvasive bioluminescence imaging to monitor the intraperitoneal growth of luciferase-transfected CC531 colorectal cells in adult WAG/RIJ rats. The bioluminescence signal correlated well with post-mortem assessment of tumour load by visual inspection of the peritoneal cavity at specific follow-up times. Using bioluminescence imaging, we were able to monitor peritoneal tumour growth sequentially in time and to calculate a tumour growth rate for each animal; this is not possible with invasive methods of evaluating tumour load. Bioluminescence imaging of rats treated with a single dose of cisplatin (4 mg kg^−1^, i.p.) demonstrated a significant delay in peritoneal tumour growth relative to saline controls (mean 45.0±s.d. 13.0 *vs* 28.2±10.3 days; *P*=0.04). Similar protocols evaluated by visual scoring of tumour load at 40 days after inoculation supported these findings, although no quantitative assessment of treatment-induced growth delay could be made by this method. This study shows that *in vivo* imaging of luciferase-transfected tumour cells is a useful tool to investigate the dynamics of disseminated tumour growth and efficacy of anticancer treatment in orthotopic models of peritoneal cancer in rats. It offers an attractive alternative to invasive methods, and requires fewer animals for measuring tumour response to therapy.

Locally disseminated peritoneal surface malignancy is a major problem in the management of abdominal cancers. In all, 10% of patients with primary colorectal cancer initially present with peritoneal seeding and 25–35% of patients who underwent seemingly curative surgery will later develop peritoneal metastases (Swedish Rectal Cancer Trial, 1997). Once peritoneal spread has occurred, survival is only 10–12 months, despite systemic chemotherapy. There is therefore an urgent need for improvements in treatment. An aggressive treatment modality currently under investigation for the treatment of colorectal cancer combines cytoreductive surgery with *H*yperthermic *I*ntraoperative *Pe*ritoneal *C*hemotherapy (HIPEC), followed by systemic chemotherapy. The aim with HIPEC is to achieve high local drug concentrations in direct contact with small peritoneal tumour deposits, with mild hyperthermia used to increase drug penetration. This technique was pioneered by Spratt *et al* (1980) and has since been evaluated in several clinical studies (Sugarbaker *et al*, 1996; Ceelen *et al*, 2000). A randomised phase III trial recently showed a significant survival benefit for HIPEC compared with systemic chemotherapy alone (Witkamp *et al*, 2001; Verwaal *et al*, 2003). However, it is not clear what effect each component of the HIPEC treatment (surgery, local perfusion, hyperthermia) has on the tumour response. Animal studies are required to identify the relative importance of different components of such multimodality therapies and to compare the effects of chemotherapy delivered by different routes.

Until now, the most commonly used method to determine tumour load in the abdominal cavity of animals is to kill the animals and score the size and number of tumours at various times after treatment (Los *et al*, 1989; Rose *et al*, 1996). Using such an approach, different animals have to be killed at sequential time points to determine the average course of tumour development and growth. This leads to the use of a large number of animals and gives an estimate of the mean tumour size, in different groups of animals, at different follow-up times. It is impossible to follow the kinetics of tumour growth and response to treatment in individual animals using such an invasive procedure. In addition, the precise tumour burden at the time of treatment is unknown, leading to uncertainty in the evaluation of treatment efficacy.

Recently, a new method of noninvasive bioluminescence imaging has been developed for *in vivo* use (Contag *et al*, 1997). Bioluminescence imaging is based on the expression of luciferase, the light-emitting enzyme of the firefly *Photinus pyralis* (De Wet *et al*, 1987). After the administration of the substrate luciferin, an ATP- and O_2_-dependent photochemical reaction occurs, resulting in the release of photons by living cells containing luciferase. This photon emission can be detected by a cooled charge-coupled device (CCD) camera, minutes after the administration of the substrate. Bioluminescence imaging has been used successfully for monitoring tumour growth in mice (Sweeney *et al*, 1999; Edinger *et al*, 1999; Vooijs *et al*, 2002; Wetterwald *et al*, 2002). There are also two reports of bioluminescence imaging in rats, to detect localised glioma growth in the brain (Rehemtulla *et al*, 2000), or to detect cardiac reporter gene expression in the heart (Wu *et al*, 2002). There are, to our knowledge, no previous reports using bioluminescent imaging to detect multifocal orthotropic tumour growth in the rat peritoneal cavity.

The aim of the present study was to determine whether bioluminescence detection offers advantages (eg increased sensitivity or reduced number of animals required to demonstrate an effect) for quantitative assessment of anticancer therapies directed against disseminated peritoneal colorectal cancer. We report on the noninvasive, *in vivo* imaging of the intraperitoneal growth of luciferase-transfected colorectal cancer cells in rats. This new method of monitoring the kinetics of tumour growth was compared with post-mortem scoring of tumour load after intraperitoneal treatment with cisplatin.

## MATERIAL AND METHODS

### Cells

Rat colorectal CC531 cells (Zedeck and Sternberg, 1974) were maintained in DMEM with 10% foetal calf serum (FCS) (Gibco, Breda, the Netherlands), penicillin (100 U ml^−1^) and streptomycin (100 U ml^−1^). These cells give rise to disseminated peritoneal tumour nodules after i.p. injection in syngeneic WAG/RIJ rats (Los *et al*, 1989).

### Animals

Female WAG/RIJ rats, bred at the animal department of the Netherlands Cancer Institute, weighing 180–200 g (age 3–5 months), were used in this study. All protocols were approved by the local Animal Experimentation Committee and carried out in compliance with national guidelines for the care and use of research animals.

### Tumour response experiments

Rats were inoculated i.p. with CC531 cells (1 × 10^6^) and treated at 10 days after tumour cell inoculation, by which time small (1–2 mm) peritoneal tumours were established at 1 or more peritoneal sites. Treatment groups consisted of a single i.p. bolus injection of cisplatin (Vianex S.A., New Erythrea, Greece; 4 mg kg^−1^ in 20 ml saline) or a 90 min intraperitoneal perfusion at 40 or 37°C with or without cisplatin (200 ml at 8 *μ*g ml^−1^). Previous studies in rats had demonstrated that approximately 30–35% of the cisplatin administered during such a 90 min peritoneal perfusion was absorbed systemically (Zeamari *et al*, 2003). The total absorbed cisplatin dose in the perfused rats was therefore slightly lower than in the bolus injection regimes (approximately 3 mg kg^−1^), but higher perfusate concentrations were associated with renal toxicity and were poorly tolerated. Animals were killed and the intraperitoneal tumour load was determined at 40 days after inoculation. Tumour load was scored according to a point scale indicating large (>5 mm: 3 points), moderate (1–5 mm: 2 points), small (<1 mm: 1 point) or no tumour (0 points) in seven abdominal regions (pelvis, omentum, transverse colon, mesentery, left and right subdiaphragmatic area, subhepatic area). These scores were added to give the total peritoneal tumour load (maximum score 21 arbitrary units).

### Intraperitoneal perfusion

Rats were anaesthetised by i.m. injection of a mixture of Hypnorm (fentanyl: 0.15 mg kg^−1^; fluanosine: 5 mg kg^−1^) and Dormicum (midazolan, 2.5 mg kg^−1^). Prior to perfusion, animals were injected i.v. with 42 mg kg^−1^ gelofusin (B. Braun, Melsungen, Germany) to increase the resistance of the veins to the pressure of the peritoneal perfusion fluid, as described previously (Zeamari *et al*, 2003). A median incision was made in the abdominal wall and the skin was sutured to a retractor ring above the abdomen, to create a basin for the perfusion fluid. Inflow and outflow catheters were introduced through the abdominal wall and connected to a heat exchanger and a regulated vacuum reservoir, as described previously (Zeamari *et al*, 2003). The perfusion system was filled with 200 ml isotonic dialysis fluid (Dianeal® PD1; Baxter, Unterscleissheim, Germany) and perfusion continued for 90 min after reaching the required temperature (37 or 40°C). Cisplatin was added to the system to achieve the required drug concentration (8 *μ*g ml^−1^) at the start of the timed perfusion. At the end of the 90 min perfusion, the perfusate was aspirated and the wound was closed.

### Luciferase transfections

CC531 cells were cotransfected with the firefly luciferase gene (pGL3) in combination with a vector for puromycin resistance (pHA262pur) or for green fluorescent protein (GFP) and neomycin resistance, using lipofectin reagent (Life Technologies, USA) or electroporation, respectively. Molar ratios of 10 : 1 were used for luciferase : selection plasmids.

At 24 h before transfection with lipofectin, cells were seeded at a density of 2 × 10^5^ per six-well plate. A total of 1 *μ*g vector DNA was then added to 100 *μ*l Optimem medium (Gibco, Breda, Netherlands) and incubated for 30 min at room temperature before mixing with 100 *μ*l lipofectin solution (2–20 *μ*l in 100 ml Optimem) and incubating for an additional 15 min. Plated cells were washed and 0.2 ml of the DNA/lipofectin mixture and 1.8 ml serum-free DMEM were added prior to overnight incubation at 37°C. Transfection medium was then replaced by fresh DMEM, containing 10% FCS, antibiotics and puromycin (5 *μ*g ml^−1^). The resulting colonies were harvested and cultured independently to select for the highest luciferase activity.

Electroporation transfections were carried out using a Genepulser equipped with an RF module (Bio-rad). A total of 4 million cells were suspended in 950 *μ*l of HEPES-buffered mannitol buffer. Luciferase and GFP plasmid (10 *μ*g) (EGFP-N3, Clontech) were added to the cell suspension and incubated for 5 min at room temperature prior to electroporation at 350 V. Cells were then incubated at 37°C for 48 h before selection by incubation with 800 *μ*g ml^−1^ neomycin (Genetecin; Gibco, Breda, Netherlands).

### Biochemical luciferase assay

Luciferase activity was measured using the Dual Luciferase Assay kit (Promega, Madison, USA). Cell suspensions were made with approximately 10^5^ luciferase-transfected cells in 100 *μ*l lysis buffer. Protein concentration was measured using pyrogallol total protein reagent (Instruchemie, Hilversum BV, Netherlands) according to the manufacturer's instructions. Luciferin substrate (50 *μ*l) was added to 5 *μ*l aliquots of cell lysates to determine the luciferase enzyme activity, using a Top Count Luminescence Counter (Perkin-Elmer Life Sciences, Boston, USA). Each lysate was measured twice and luciferase activity, corrected for protein content, was expressed as relative light units (RLUs).

### *In vivo* bioluminescence imaging

All animals were imaged using a cooled CCD camera (Xenogen IVIS, Xenogen, Alameda, USA), coupled to the LivingImage acquisition and analysis software (Xenogen Corp.). To determine the detection limits of CC531-luc cells in rats, an increasing number of tumour cells was initially inoculated within the abdominal wall, immediately followed by the injection of luciferin and imaging of photon emission after 10 min. On the basis of this result, images were subsequently made at various time points after i.p. inoculation of 2 × 10^6^ CC531-luc cells to monitor the dynamics of peritoneal tumour growth.

Before imaging, the abdomen of the rats was shaved to minimise photon scattering and signal quenching. D-luciferin (Xenogen) was dissolved at 15 mg ml^−1^ in sterile PBS and stored at −20°C. Animals were anaesthetised with hypnorm (fentanyl: 0.15 mg kg^−1^; fluanosine: 5 mg kg^−1^) and Dormicum (midazolam: 2.5 mg kg^−1^) prior to injection with luciferin (150 *μ*g g^−1^ body weight, i.p.). Peritoneal light emission was measured over an integration time of 1 min, and images were acquired using Ivis and LivingImage software. Signal intensity was quantified as the total counts measured over the region of interest.

One group of rats (*n*=5) was followed sequentially at weekly intervals from 1 to 5 weeks after inoculation and then killed for visual inspection of peritoneal tumour load. Separate groups of rats (*n*=5 or 6 per time point) were imaged at 1, 2, 3 or 4 weeks immediately prior to post-mortem evaluation by visual inspection. Peritoneal luciferase activity was correlated with the distribution and size of tumours determined using the scoring system described.

### Bioluminescence imaging of effect of i.p. cisplatin treatment on tumour growth

Rats were inoculated with 2 × 10^6^ CC531-luc cells i.p. prior to sequential imaging at weekly intervals. These images showed that the majority of animals had a detectable luciferase signal after 1 week. Animals with detectable signal were assumed to be tumour bearing and were subsequently treated on days 8–10 with either cisplatin (4 mg kg^−1^ in 20 ml saline, i.p.), saline only (20 ml, i.p.) or no treatment (*n*=5–7 per group). Luciferase activity was imaged weekly (for a maximum of 11 weeks) to determine the effect of the treatment on tumour growth. Animals were killed when the peritoneal RLU count was >2000 × 10^3^.The time taken for peritoneal RLU signal to increase by 1000 counts above the starting value (on days 8–10 after inoculation) was calculated for each rat; this was termed ‘tumour regrowth time’.

## RESULTS

### Effect of cisplatin treatment on intraperitoneal tumour growth

**Figure 1:**
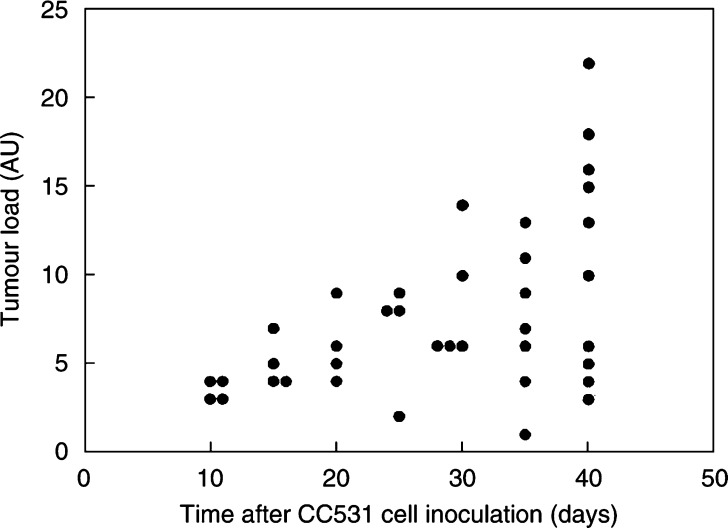
Intraperitoneal tumour load in individual rats at 10 to 40 days after inoculation of 1 × 10^6^ CC531 cells. The tumour load (in arbitrary units) was estimated by visual inspection at post-mortem, according to a point scale combining number and size of nodules (see Materials and methods).

CC531 cells grew intraperitoneally in WAG/RIJ rats and induced superficial tumours at several sites in the abdominal cavity, including omentum, mesentery, pelvis and subhepatic and subdiaphragmatic area. However, there was a large variation in tumour load within each group of animals killed at different times after inoculation (Figure 1

). At 40 days after inoculation, the tumour load ranged from 1 or 2 small nodules (a point score of 3) to extensive multifocal disease throughout the peritoneal cavity (score 21). Since this tumour scoring system is only semiquantitative, and different rats were examined at each time point, it is not possible to calculate tumour volume doubling times or growth rates from these data.

**Figure 2:**
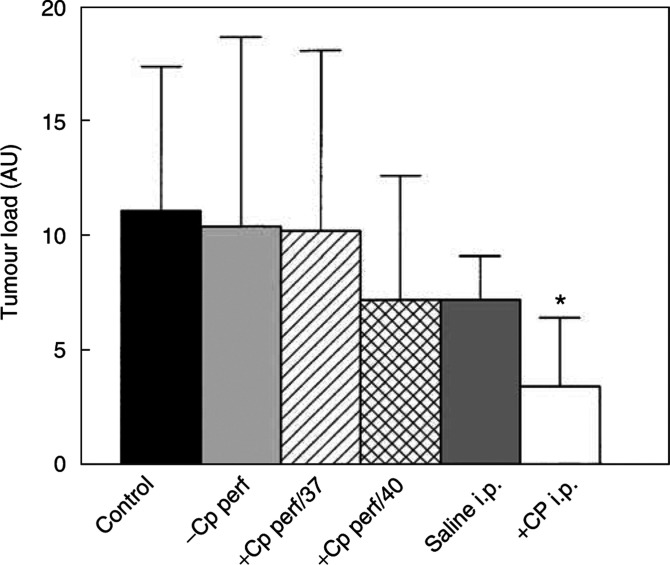
Mean tumour load (±s.d.) estimated for groups of treated (*n*=5–7) and untreated rats (*n*=11) at 40 days after inoculation with CC531 cells. Control rats received no surgical treatment or drugs; −CP perf=90 min perfusion at 37°C with 200 ml Dianeal perfusate only; +CP perf/37=90 min perfusion at 37°C with cisplatin in 200 ml Dianeal (8 *μ*g ml^−1^); +CP perf/40=90 min perfusion at 40°C with cisplatin in 200 ml Dianeal (8 *μ*g ml^−1^); saline i.p.=bolus injection of 20 ml saline (i.p.); +CP i.p.=bolus injection of cisplatin (4 mg kg^−1^) in 20 ml saline (i.p.). Tumour load for cisplatin bolus injection was significantly lower than for i.p. saline controls (^*^).

Separate groups of rats were treated with cisplatin given either as an i.p. bolus injection (4 mg kg^−1^ in 20 ml saline) or intra-abdominal perfusion at 37 or 40°C (8 *μ*g ml^−1^ in 200 ml Dianeal), 10 days after inoculation with CC531 cells. The tumour load was assessed post-mortem at 40 days after inoculation (Figure 2

). These results show a significant reduction in tumour load after cisplatin given as an i.p. bolus injection (*P*=0.03, relative to saline controls). There was no reduction in tumour load after normothermic or hyperthermic perfusion with cisplatin, relative to perfuate-only controls (*P*>0.5). The i.p. bolus cisplatin treatment protocol was subsequently selected for evaluating the usefulness of bioluminescence imaging to detect tumour response to therapy.

### Luciferase expression in CC531cells

The highest *in vitro* luciferase activity detected in CC351 transfected cells was 4.2 × 10^5^ counts mg^−1^ protein after lipofectin transfection (CC531-luc_1_) and 2.9 × 10^3^ counts mg^−1^ after electoporation (CC531-luc_2_). There was no difference in the *in vitro* growth of CC531 parental cells and CC531-luc_1_ or -luc_2_ cells, indicating that the transfection procedure had no effect on the *in vitro* growth ability (data not shown). These two cell lines were used for further *in vivo* studies.

Both the transfected cell lines CC531-luc_1_ and CC531-luc_2_ produced tumours in immune suppressed nude mice when injected subcutaneously or i.p. However, only the electroporation- transfected CC531-luc_2_ cells grew in immune competent WAG/RIJ rats. The detection limit for CC531-luc_2_ cells, imaged immediately after injection in the rat abdominal wall, was 10^5^ cells.

**Figure 3:**
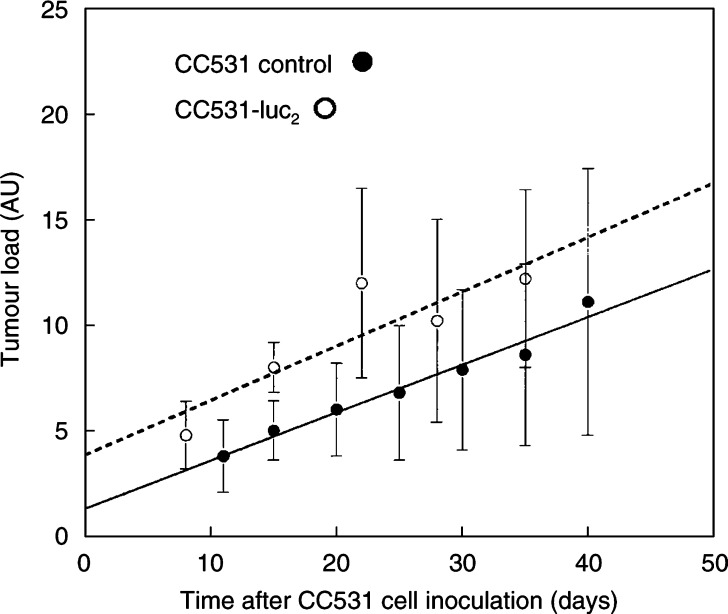
Mean tumour load (±s.d.) estimated, by visual inspection at post-mortem, for groups of rats at various times after inoculation with 1 × 10^6^ CC531 cells (•), or 2 × 10^6^ CC531-luc_2_-transfected cells (○). Groups comprise four to five rats, or seven to 10 rats (CC531 cells at 35 and 40 days).

The first peritoneal tumour nodules were visible by manual inspection about 1 week after i.p. inoculation of CC531-luc_2_ cells; that is, earlier than after i.p. inoculation of rats with the parental CC531 cells. This reflects the change in protocol to inject 2 × 10^6^ transfected cells compared with 1 × 10^6^ parental cells. However, there was no significant difference in the *in vivo* growth rates of CC531-luc_2_ cells compared with the parental cells; the mean tumour loads increased from about 5 to 12 arbitrary units over a 4-week period (Figure 3

).

### Bioluminescence imaging of tumours in animals

**Figure 4:**
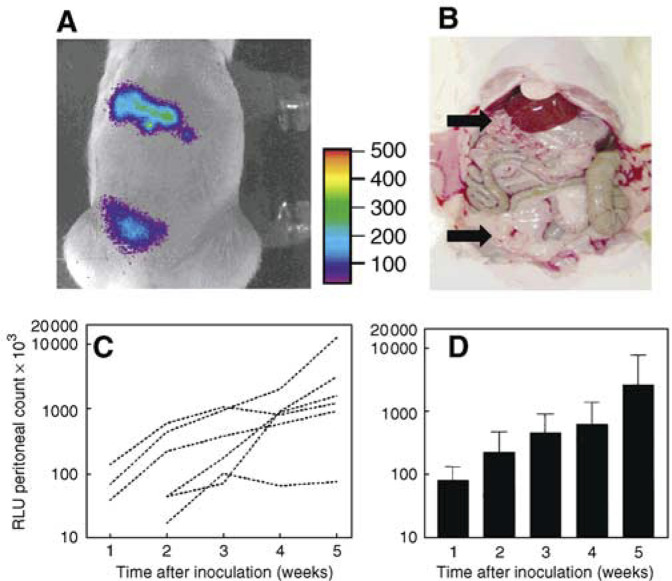
Correlation of external bioluminescence imaging of rats (**A**) with location of tumour (**B**) at 21 days after inoculation of CC531- luc_2_ cells. The scale to the right of the image (**A**) describes the colour map for the luminescence signal (RLU). **C**) shows increasing bioluminescence signals in six rats imaged repeatedly from 1 to 5 weeks after tumour cell inoculation. Group mean values (±s.d.) for these rats are shown in (**D**) (*n*=3 at 1 week, *n*=6 at other times).

Intraperitoneal growth of CC531-luc_2_ tumour cells was sequentially monitored in a group of five rats, using the total light emission over the peritoneal cavity as an indication of tumour burden. Peritoneal luciferase activity could be detected in rats from 1 to 2 weeks after inoculation of 2 × 10^6^ CC531-luc_2_ cells. The peritoneal tumour distribution, estimated by visual inspection at the time of killing, correlated with the regions of bioluminescence activity (Figures 4A and B

). Relative light units increased with time after inoculation (Figure 4C and D), and the time taken for peritoneal luminescence scores to increase from 100 to 1000 × 10^3^ counts ranged from 2 to >5 weeks.

**Figure 5:**
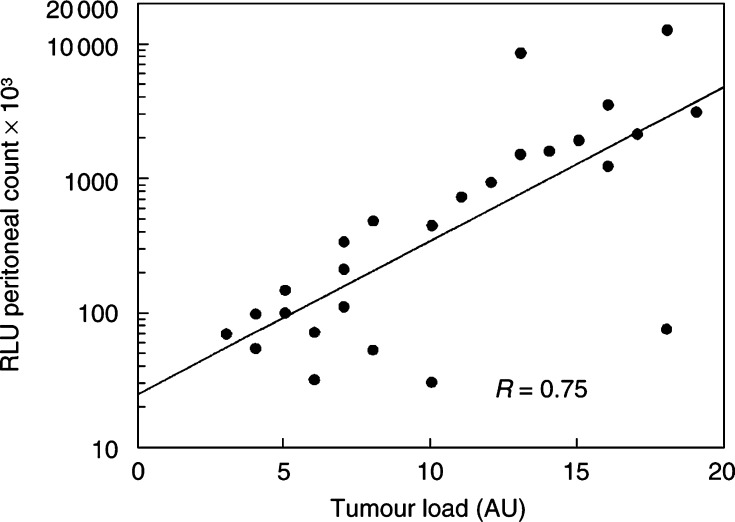
Correlation between bioluminescence signal (measured immediately before killing) and post-mortem evaluation of tumour load (*r*=0.75). Data are for individual, untreated rats at 1–5 weeks after CC531-luc_2_ cell inoculation.

Separate groups of tumour-bearing rats were also imaged and then killed to estimate tumour load at 1, 2, 3 and 4 weeks after inoculation. There was a good overall correlation (*r*=0.75) between bioluminescence imaging and tumour load for individual rats (Figure 5

). However, there was one rat with obvious tumour nodules visible at post-mortem that were not detected by bioluminescence. This rat had tumours in the subhepatic region, which were shielded by the liver and stomach; nodules in other regions were <2 mm.

### Response to chemotherapy *in vivo*

**Figure 6:**
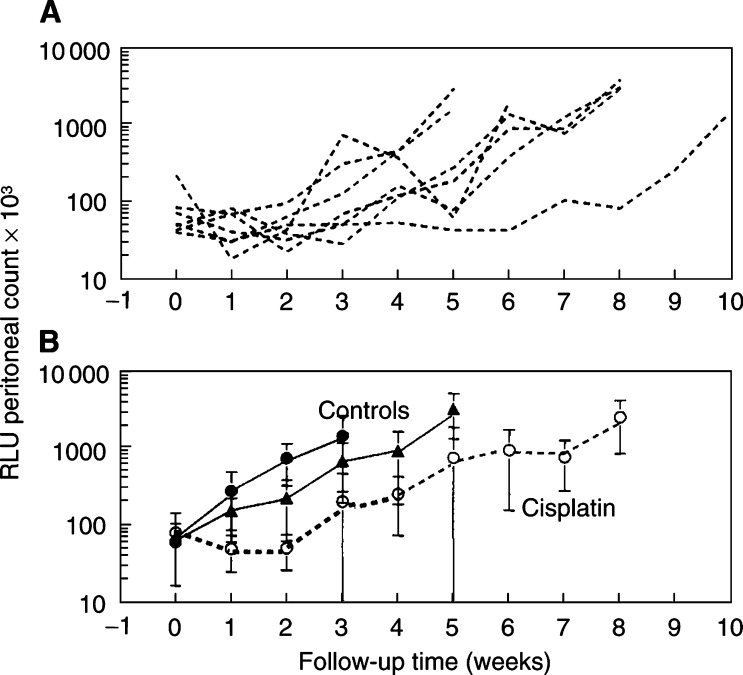
Bioluminescence signals in seven individual rats imaged at weekly intervals after an i.p. bolus injection of cisplatin (**A**). The group mean values (±s.d.) for these rats (○) are compared with saline controls (*n*=7, ▴) and untreated rats (*n*=5, •) in (**B**).

**Figure 7:**
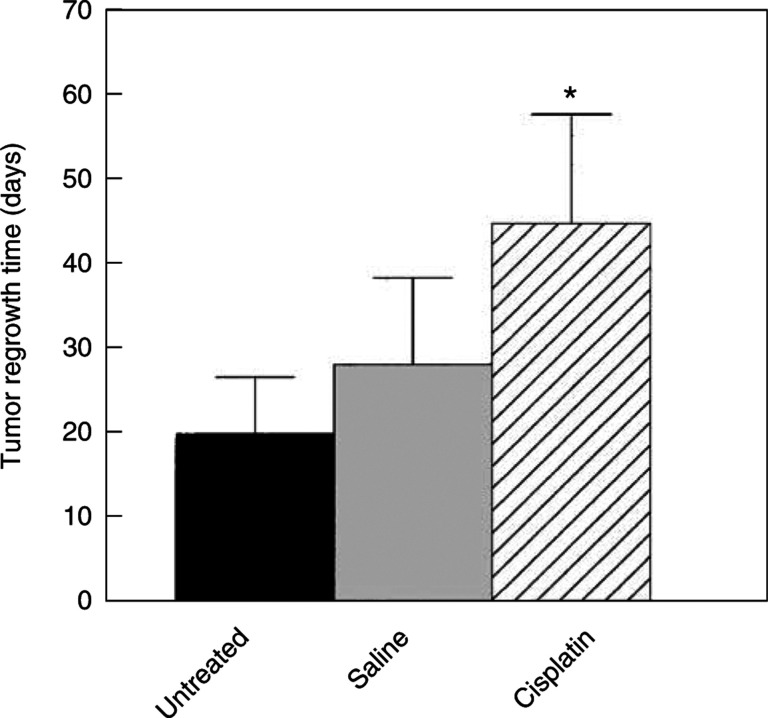
Tumour regrowth times (mean±s.d.) for cisplatin-treated (*n*=7) and control rats (*n*=5–7), calculated as the time taken for the RLU signal to increase by 1000 counts above the value at treatment (>40 RLU). Cisplatin-treated rats had significantly longer regrowth times (^*^) than untreated (*P*=0.003) or saline-treated rats (*P*=0.038). There was no significant difference between untreated and saline controls.

*In vivo* imaging of luciferase activity of CC531-luc_2_ cells was subsequently used to determine the effect of i.p. bolus injection with cisplatin on intraperitoneal tumour growth in WAG/RIJ rats (Figure 6

). Four of the seven cisplatin-treated rats clearly had slower progression of their peritoneal disease than untreated rats, or rats given a bolus injection of saline. Tumour progression was unchanged in two rats and one rat had variable RLU signals from week to week. The group mean tumour regrowth time (time required for the bioluminescence signal to increase by 1000 RLU) for cisplatin-treated rats was significantly longer than that for saline-treated rats rats: 45.0±13.1 (s.d.) *vs* 28.2±10.3 days (*P*=0.038), or for untreated rats (19.0±6.8 days, *P*=0003). There was no significant difference between the untreated control group and the group given i.p. saline (Figure 7

).

## DISCUSSION

Sensitive and noninvasive methods for measuring tumour load are needed to evaluate and compare the efficacy of different cancer treatments *in vivo*. Our study demonstrates, for the first time, that *in vivo* bioluminescence imaging of luciferase-transfected cells can be used for the effective assessment of disseminated peritoneal tumour growth and response to treatment in rats, thereby extending its use beyond imaging of focal superficial tumours. Tumour nodules of 2–3 mm could be detected in the peritoneal cavity, providing thick, pigmented organs, like the liver, did not shield them. This technique therefore offers an attractive alternative to more commonly used invasive methods, in which groups of animals are killed and tumour load estimated by visual inspection or excision and weighing at different time points after treatment (Los *et al*, 1989; Rose *et al*, 1996).

As part of our ongoing investigation to compare the efficacies of various treatments against disseminated peritoneal cancer, we initially used visual, post-mortem inspection of tumour load at various times after inoculation with CC531 cells. This revealed substantial differences between the rats. Such a variation in tumour growth rate inevitably resulted in a wide range of tumour burdens in animals treated at a prespecified time after inoculation. Despite this variation, we were able to demonstrate a significant reduction in tumour load after treatment with a bolus injection of cisplatin at 4 mg kg^−1^, close to the MTD, although no reduction in tumour burden was seen for perfusion with cisplatin at MTD (8 *μ*g ml^−1^). Previous studies demonstrated that peritoneal perfusion with 15 *μ*g ml^−1^ cisplatin (at 37 or 40°C) was required to give the same tumour concentration as an i.p. bolus injection of 4 mg kg^−1^ (Zeamari *et al*, 2003). These schedules also resulted in similar plasma drug levels, which suggests that the drug concentration in small peritoneal tumours was largely determined by systemic exposure, rather than peritoneal drug concentrations. In the present study, the concentration of cisplatin in the perfusate had to be reduced, in order to avoid excessive renal toxicity. This must have resulted in lower tumour drug concentrations than the i.p. bolus injections (although these were not measured in the present study), which is probably the reason for the lack of efficacy for the perfusion schedules used. These observations, although not conclusive, do not lend any support to the proposals for an improved therapeutic ratio for hyperthermic, peritoneal, perfusion with low doses of cisplatin.

To overcome the disadvantages of invasive monitoring of tumour growth, we subsequently used *in vivo* bioluminescence imaging of luciferase-transfected CC531 cells. We started with CC531-luc_1_ cells, transfected using lipofectin reagent. This gave good luciferase activity and we were able to monitor the intraperitoneal growth of these CC531-luc_1_ cells in nude BALB/c mice by external, bioluminescence imaging. These CC531-luc_1_ cells were, however, not able to form tumours after inoculation in WAG/RIJ rats. The reason for this was probably immunogenicity induced by the transfection process. Parental CC531 cells were subsequently transfected using electroporation, which resulted in a much lower level of luciferase gene expression (by a factor of >100) compared to lipofectin transfection. These CC531-luc_2_ cells did grow in the peritoneal cavity of the WAG/RIJ rats and no significant differences between growth rates of parental and transfected cells were seen. The high level of transfected gene expression in CC531-luc_1_ cells could have been responsible for their inability to form tumours in immunocompetent rats. It has been previously reported that expression of high levels of a transfected gene, for example, GFP, in mouse BM185 leukaemia cells resulted in a drastic reduction in disease development in immunocompetent mice, whereas in immunodeficient Nu/Nu mice the disease developed rapidly (Stripecke *et al*, 1999).

Using *in vivo* imaging of luciferase-transfected cells, longitudinal data could be acquired, monitoring the intraperitoneal distribution and growth of tumour cells with time after inoculation. This also allowed us to specify a minimum tumour burden at the time of treatment and a maximum tumour burden prior to killing the animals. Neither of these can be done when tumour load is evaluated by invasive methods at specific, preplanned times. Comparison of the tumour load evaluated by bioluminescence and post-mortem visual inspection revealed good correlations (*r*=0.75) with regard to both the extent and distribution of tumour, despite the multiple tumour foci at variable depth in the peritoneal cavity. Small tumours within the liver mass could not be easily detected by bioluminescence.

Bioluminescence imaging was used to monitor responses to a bolus, i.p. injection of cisplatin. Early response (tumour regression) was followed by subsequent regrowth of tumours. This could be detected well before the appearance of the common end points of weight loss, palpable tumours or death. A significant effect of cisplatin treatment on tumour growth, determined by *in vivo* bioluminescence, has already been reported in mice (Sweeney *et al*, 1999). However, this is the first study that shows such an effect in rats, using a bioluminescence technique.

Despite the advantages and convenience of bioluminescence imaging for monitoring tumour growth, there are some disadvantages. Firstly, light transmission is attenuated by tissue; therefore the deeper the tumours lie within the body, the greater the signal attenuation. This means that tumour load from deeper parts of the body will be relatively under-represented in the integrated images. It also means that small tumours that are shielded by bulky, pigmented organs may well be missed. Light–coloured organs, like prostate and mammary tissue or lungs, will tend to scatter rather than absorb the light, so the bioluminescence signal from tumour cells within such organs should be much less attenuated.

A second disadvantage of bioluminescence detection is that this procedure involves transfection of the parental cell line. The resulting clones do not always behave in exactly the same way as the parental cells, as was demonstrated by the inability of CC531-luc_1_-transformed cells to grow in immunocompetent rats. Ideally, one would like to use clones expressing very high levels of the reporter gene, since this will enhance detection sensitivity. However, high expression levels of the transfected gene are also the most likely to introduce unwanted phenotypic changes, for example, immunogenicity and altered growth rates. In reality, a compromise between high luciferase expression and no, or minimal, changes in tumour cell behaviour will probably need to be sought. In any case, each transfected cell line should be checked for altered tumour growth before use in evaluating treatment responses.

Alternative noninvasive techniques, such as PET, might overcome some of the limitations of bioluminescence detection of orthotropic tumour growth. However, this is a very expensive technique and it is not widely available for preclinical animal studies. PET may also be associated with its own problems, since the radioisotopes used tend to accumulate in excretion organs (liver, kidneys, bladder), which could interfere with the detection of small tumours in the peritoneal cavity. A direct comparison of these techniques would be interesting, but is outside the scope of the present study.

In summary, we showed that *in vivo* bioluminescence imaging of tumour cells enables a rapid, noninvasive measurement of tumour load before, during and after treatment of rats with disseminated peritoneal disease. Using this technique, our understanding of *in vivo* tumour development and response to current and novel treatments can be increased.
